# Aspirin relieves the calcification of aortic smooth muscle cells by enhancing the heat shock response

**DOI:** 10.1080/13880209.2021.2007268

**Published:** 2021-11-30

**Authors:** Quanquan Shen, Qian Chen, Yang Liu, Xiang Xue, Xiaogang Shen, Qiang He, Guokun Wang, Fei Han

**Affiliations:** a Kidney Disease Center, The First Affiliated Hospital, Zhejiang University School of Medicine, Hangzhou, China; b Institute of Nephrology, Zhejiang University, Hangzhou, China; c Key Laboratory of Kidney Disease Prevention and Control Technology, Hangzhou, China; d Department of Nephrology, Zhejiang Provincial People’s Hospital, Affiliated People’s Hospital, Hangzhou Medical College, Hangzhou, China; e Department of Cardiovascular Surgery, Institute of Cardiac Surgery, Changhai Hospital, Naval Medical University, Shanghai, China

**Keywords:** Vascular smooth muscle cells, heat shock protein 70, heat shock protein 90, heat shock transcription factor 1

## Abstract

**Context:**

Vascular calcification is a major complication of chronic renal failure, which has been identified as an active process partly driven by osteogenic transition of vascular smooth muscle cells (VSMCs). Aspirin could prevent cardiomyocyte damage by inducing heat shock response.

**Objective:**

This study investigates the effect of aspirin on alleviating VSMC calcification.

**Materials and methods:**

An *in vitro* VSMC calcification model was established by 10-day calcification induction in osteogenic medium. VSMCs were grouped as following: control group (normal medium), calcified group (osteogenic medium) and treated group (osteogenic medium with 1 or 4 mmol/L aspirin). VSMC calcification was evaluated by calcified nodules formation, intracellular calcium concentration and osteoblastic marker (OPN and Runx2) expression.

**Results:**

After 10-day culture, the intracellular calcium concentration in calcified group was significantly higher than that in control group (1.16 ± 0.04 vs. 0.14 ± 0.01 μg/mg, *p* < 0.01), but significantly reduced in 1 mmol/L aspirin treated group (0.74 ± 0.05 μg/mg, *p* < 0.01), and 4 mmol/L aspirin treated group (0.93 ± 0.03 μg/mg, *p* < 0.01). The elevated expression of OPN and Runx2 induced by osteogenic medium was significantly relieved after 1 or 4 mmol/L aspirin treatment. The expression of HSF1, HSP70 and HSP90 was decreased in calcification-induced VSMCs, but significantly increased after treatment of aspirin. Furthermore, inhibition of HSP70 (or HSP90) by small-molecule inhibitor or small interfering RNA could partially abolish the anti-calcification effect of aspirin, proved by the changes of intracellular calcium concentration and osteoblastic marker expression.

**Discussion and conclusions:**

Aspirin could relieve the calcification of VSMCs partially through HSP70- or HSP90-mediated heat shock response. These findings expanded the understanding of aspirin pharmacology, and imply that local induction expression of HSPs might be a potential therapeutic strategy for the prevention and therapy of vascular calcification.

## Introduction

Vascular calcification pathologically manifests as transition of vascular smooth muscle cells (VSMCs) into osteoblast-like cells with calcium-phosphate complexes depositing in the intimal or medial vascular wall. This process is strongly correlated to mortality and progression of cardiovascular disease (Rocha-Singh et al. 2014; Durham et al. 2018; Lee et al. 2020). Vascular calcification has been associated to several pathological factors, such as chronic kidney disease (CKD), diabetes mellitus and essential hypertension (Zhang et al. 2018). Vascular calcification may occur in the early stage of CKD and accompany CKD progression. The documented incidence rate is up to 80–90% in CKD stage 5 patients (Karohl et al. 2011). Dysregulated calcium and phosphorus metabolism and failure in anti-calcification are linked to vascular calcification in CKD patients (Paloian and Giachelli 2014). However, the specific mechanism of early onset of vascular injury and calcification remains unclear.

Heat shock proteins (HSPs) are a family of ubiquitous proteins, that rapidly respond to heat and other environmental stresses (Mehta et al. 2005; Wang et al. 2016). HSPs, such as HSP70 and HSP90, can protect cells from damage to a certain extent and improve cellular viability by maintaining the stability of protein structure and activity (Bielecka-Dabrowa et al. 2009; Doyle et al. 2019). The expression of HSPs can be rapidly induced by heat shock transcription factor 1 (HSF1), which is essential to cell-fate decisions underlying survival or death (Barna et al. 2018; Zhao Z et al. 2019). Recent studies have shown that the expression of HSPs is closely related to the process of cardiovascular calcification. The expression of HSP70 is compromised in CKD patients with coronary artery disease (CAD), and induction of intracellular HSP70 could prevent the development of VSMC calcification (Lu et al. 2012). Downregulation of HSP90 was also found in calcified aortic valves, suggesting HSP90 as a central signalling molecule in aortic valvular calcification (Weisell et al. 2019).

Acetylsalicylic acid (aspirin) is a well-known antiplatelet drug, which inhibits platelet aggregation by blocking cyclooxygenase (COX) enzymes (Scharf 2012). Clinical trials have substantiated the use of aspirin in preventing adverse events in patients with cardiovascular disease and venous thromboembolism (Weitz et al. 2017; Byrne and Colleran 2020). As a pleiotropic agent, aspirin has antioxidant, anti-inflammatory, antipyretic and analgesic effects, and has shown certain potential in tumour prevention (Desborough and Keeling 2017; Skelin et al. 2019; Zhao AS et al. 2019; Sankaranarayanan et al. 2020). Recent studies suggested that aspirin could induce the expression of HSPs in myocardial cells, and protect cardiomyocytes from the damage of heat stress (Wu et al. 2016; Zhang et al. 2016).

Here, we established an *in vitro* VSMC calcification model, and evaluated the role of aspirin on reducing VSMC calcification. We also determined the expression and role of HSPs in the process of VSMCs calcification. The results may provide an experimental basis for the potential application of aspirin in the prevention and treatment of vascular calcification.

## Materials and methods

### Primary VSMCs isolation and culture

Primary VSMCs were isolated from aortas of adult male Sprague-Dawley rats (SLAC, Shanghai, China) by collagenase digestion (Zhao Z et al. 2019). Then cells were seeded in DMEM containing 10% foetal bovine serum (FBS) and 1% penicillin–streptomycin and cultured in cell incubator at 37 °C of 5% CO_2_. All cell experiments were performed on VSMCs at passage 5. All the experimental procedures conformed to the Animal Welfare Act Guide for Use and Care of Laboratory Animals, and were approved by the Experimental Animal Ethics Committee of Hangzhou Medical College (no. 20190163).

### Immunofluorescence staining

The purity of VSMCs was identified by immunofluorescence staining of α-SMA. Briefly, 4% paraformaldehyde-fixed VSMCs were permeabilized and blocked by goat serum. Then cells were incubated in primary antibody anti-α-SMA (1:1000. Abcam, Cambridge, UK) for 12 h at 4 °C, followed by incubation in Alexa Fluor 488-conjugated secondary antibody (Thermo Fisher Scientific, Plainville, MA) for 1 h at room temperature. The nucleus was labelled by 4′,6-diamidino-2-phenylindole (DAPI). Subsequently, images were captured by inverted biologic fluorescence microscope (Olympus, Tokyo, Japan).

### VSMCs calcification induction and treatment

The calcification of VSMCs was induced by osteogenic induction medium (OIM), which was composed of 2 mmol/L NaH_2_PO_4_, 50 μg/mL vitamin C, 0.1 μmol/L insulin, 5% FBS and DMEM. Aspirin (1 mmol/L or 4 mmol/L; Sangon Biotech, Shanghai, China), HSP70 inhibitor MKT-077 (1 µmol/L; MedChemExpress, Monmouth Junction, NJ) and HSP90 inhibitor 17-DMAG (1 µmol/L; Sigma, St. Louis, MO) were applied to treat the calcification-induced VSMCs.

### VSMC calcification detection

The formation of calcified nodules was detected by Alizarin Red S staining kit (Beyotime, Shanghai, China) according to the instruction. The intracellular calcium concentration was detected by Calcium Colorimetric Assay kit (Sigma, St. Louis, MO).

### Small interfering RNA (siRNA) transfection

siRNA targeting HSF1, HSP70 or HSP90 was designed and synthesized by Ribo Biotechnology Co. Ltd. (Guangzhou, China). SiRNA transfection was performed by riboFECT CP transfection kit (Ribo Biotechnology, Guangzhou, China) according to the instruction manual.

### Western blotting

VSMCs were lysed on ice for 30 min with SDS Lysis Buffer. The concentration of acquired protein in cell lysis was measured by BCA protein quantitative kit (Thermo Scientific, Carlsbad, CA). Equal amounts of protein samples were added into the lane of SDS-PAGE gels. After electrophoresis and transmembrane, the membrane was incubated with primary antibodies and following secondary antibodies. Then, the immunoreactive bands were visualized by chemiluminescence (Thermo Scientific, Carlsbad, CA) and analysed by Image J software (Bethesda, MD). β-Actin was used as loading control. The primary antibodies included anti-OPN (1:1000 dilution, Abcam, Cambridge, UK), anti-Runx2 (1:1000 dilution, Abcam, Cambridge, UK), anti-HSF1 (1:500 dilution, Proteintech, Wuhan, China), anti-HSP70 (1:500 dilution, Boster, Wuhan, China), anti-HSP90 (1:500 dilution, Boster, Wuhan, China) and anti-β-actin (1:1000 dilution, Proteintech, Wuhan, China).

### Statistical analysis

All statistical analyses were performed by SPSS 22.0 (Chicago, IL). The difference between two groups was analysed by independent sample *t*-test (normal distribution) or Mann–Whitney’s *U* test (non-normal distribution) after normal distribution test. The difference among multiple groups was analysed by one-way ANOVA followed by post hoc Tukey’s test (homogeneity of variance) or one-way ANOVA followed by Dunnett's T3 test (heterogeneity of variance). *p* < 0.05 was considered to be statistically significant.

## Results

### Establishment and identification of an *in vitro* VSMC calcification model

**Figure 1.**
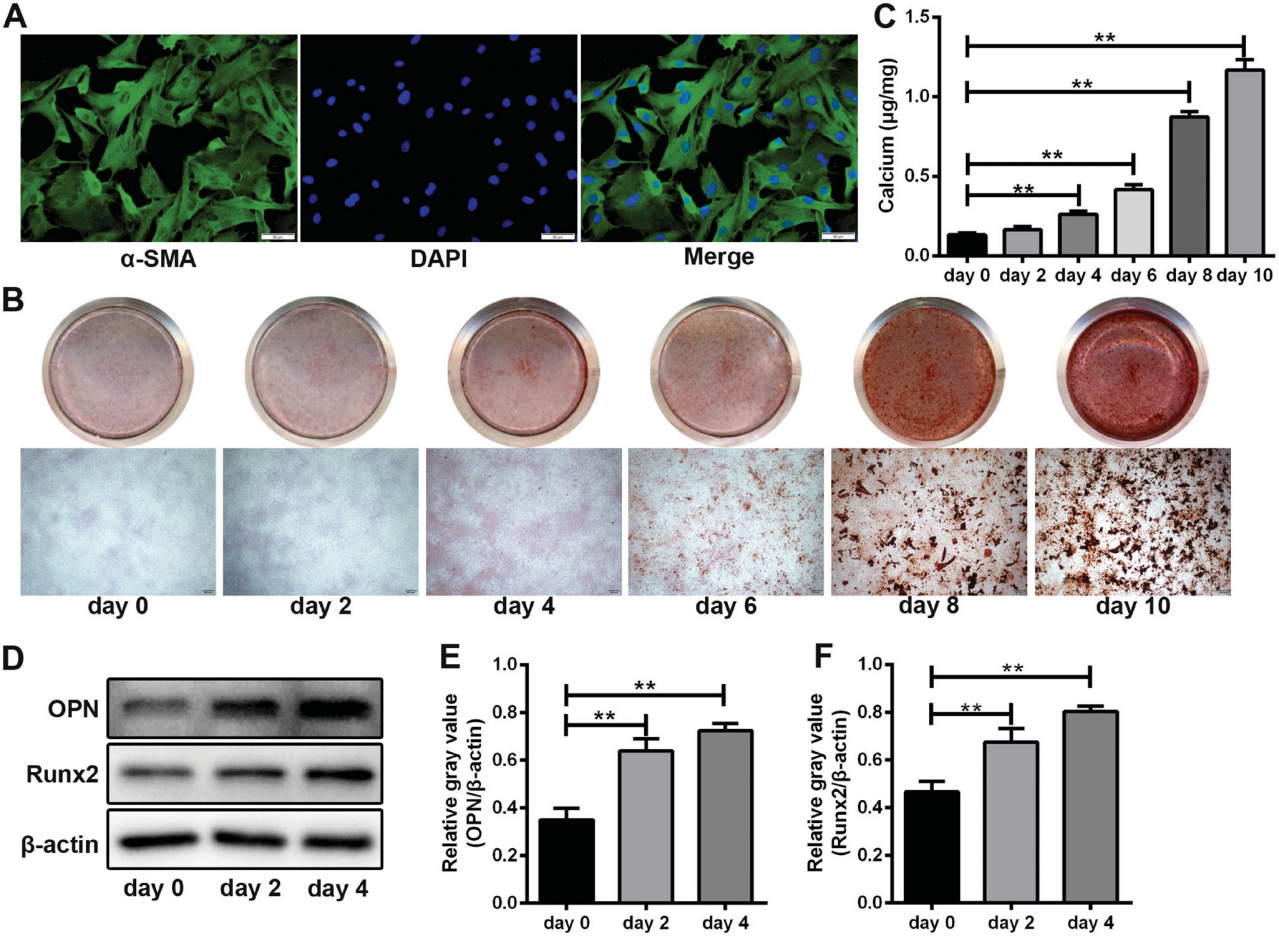
Establishment and identification of an *in vitro* VSMC calcification model. (A) Representative immunofluorescence images of α-SMA (green) in isolated aortic VSMCs. Nucleus was stained by DAPI (blue). (B) Detection of calcium nodules in VSMCs after calcification induction by Alizarin Red S assay. (C) Measurement of intracellular calcium concentration during VSMC calcification induction. *n* = 5 in each group. **p < 0.01. (D) Detection of OPN and Runx2 protein expression in VSMCs after calcification induction by western blot assay. β-Actin was used as loading control. (E, F) Grey values analysis of immune bands in western blot assay. *n* = 3 in each group. **p < 0.01.

Immunofluorescence staining assay indicated that α-SMA-positive cells in the primary VSMCs were above 90% (Figure 1(A)), indicating that the purity of extracted VSMCs was satisfactory. Then VSMCs were cultured in OIM for calcification induction in 10 days. A small number of calcium nodules emerged and the intracellular calcium concentration increased significantly on the 4th day, and the calcification process aggravated in the following days (Figure 1(B,C)). The levels of osteoblastic markers (OPN and Runx2) were significantly increased on the 2nd day and 4th day after calcification induction (Figure 1(D–F)). These results showed that the *in vitro* model of VSMC calcification was well developed.

### Aspirin alleviated the calcification progress of VSMCs

**Figure 2.**
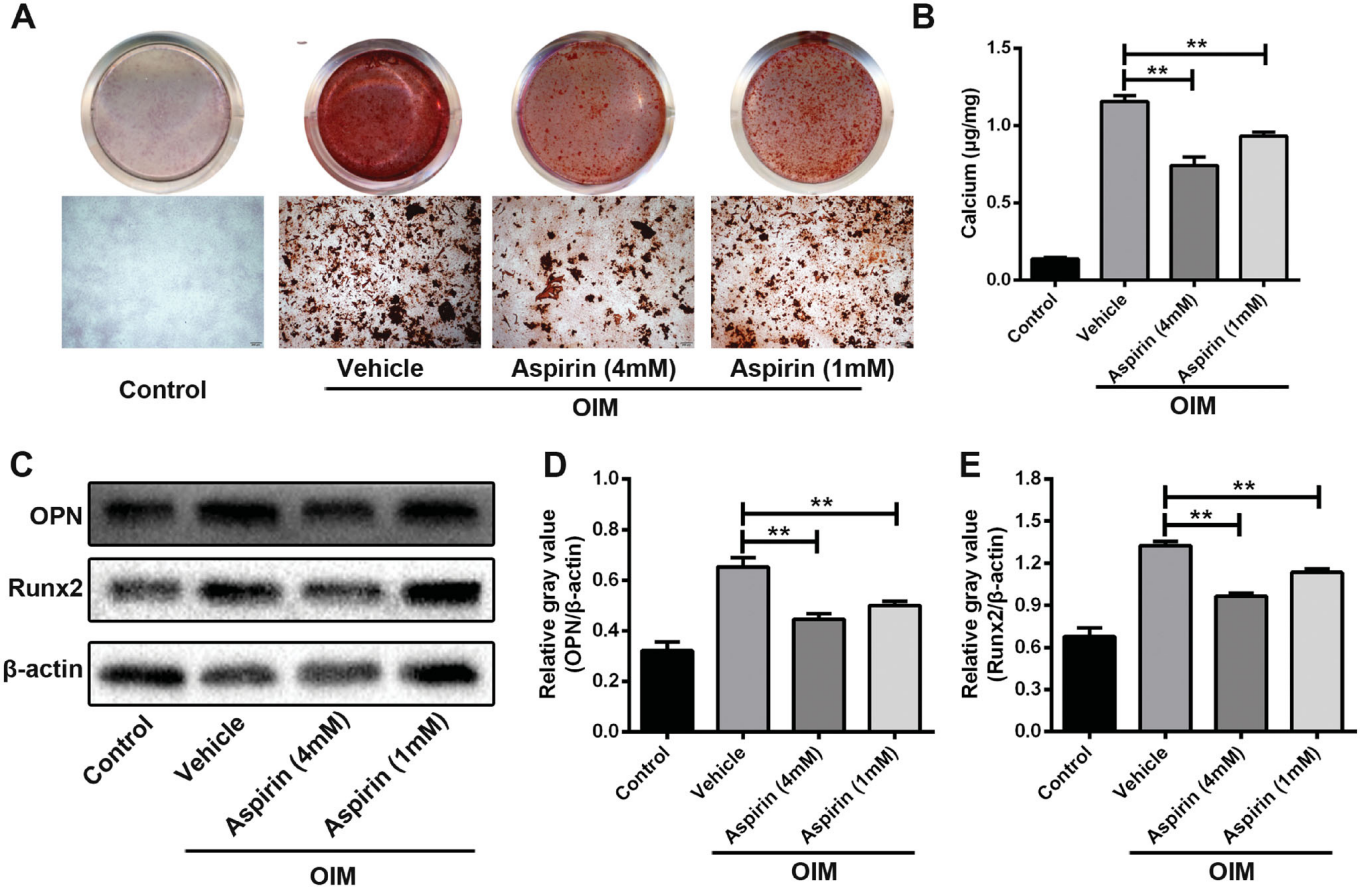
Aspirin treatment alleviated the calcification progress of VSMCs. (A) The effect of aspirin on calcium nodules in VSMCs. Alizarin Red S assay was applied to detect calcium nodules in VSMCs after 10-day culture in OIM. (B) The effect of aspirin on intracellular calcium concentration in VSMCs after 10-day culture in OIM. *n* = 5 in each group. **p < 0.01. (C) The effect of aspirin treatment on expression of osteogenic marker genes in VSMCs after four-day culture in OIM. Western blot assay was used to detected protein expression of OPN and Runx2 in VSMCs. β-Actin was used as loading control. (D, E) Grey values analysis of immune bands in western blot assay. *n* = 3 in each group. **p < 0.01.

To test the effect of aspirin on VSMC calcification, two doses of aspirin (1 and 4 mmol/L) were applied to treat VSMCs in the process of calcification induction. Compared with control group, calcification-induced VSMCs showed a mass of calcium nodules and high concentration of intracellular calcium. The formation of calcium nodules and accumulation of intracellular calcium in VSMCs were significantly decreased by aspirin treatment in both concentrations; while in 4 mmol/L aspirin group, the decrease tended to be more severe (Figure 2(A,B)). Western blot assay also showed that the OIM-induced elevated expression of OPN and Runx2 was significantly downregulated by aspirin treatment in both concentrations (Figure 2(C–E)).

### Aspirin enhanced the heat shock response in the process of VSMC calcification

**Figure 3.**
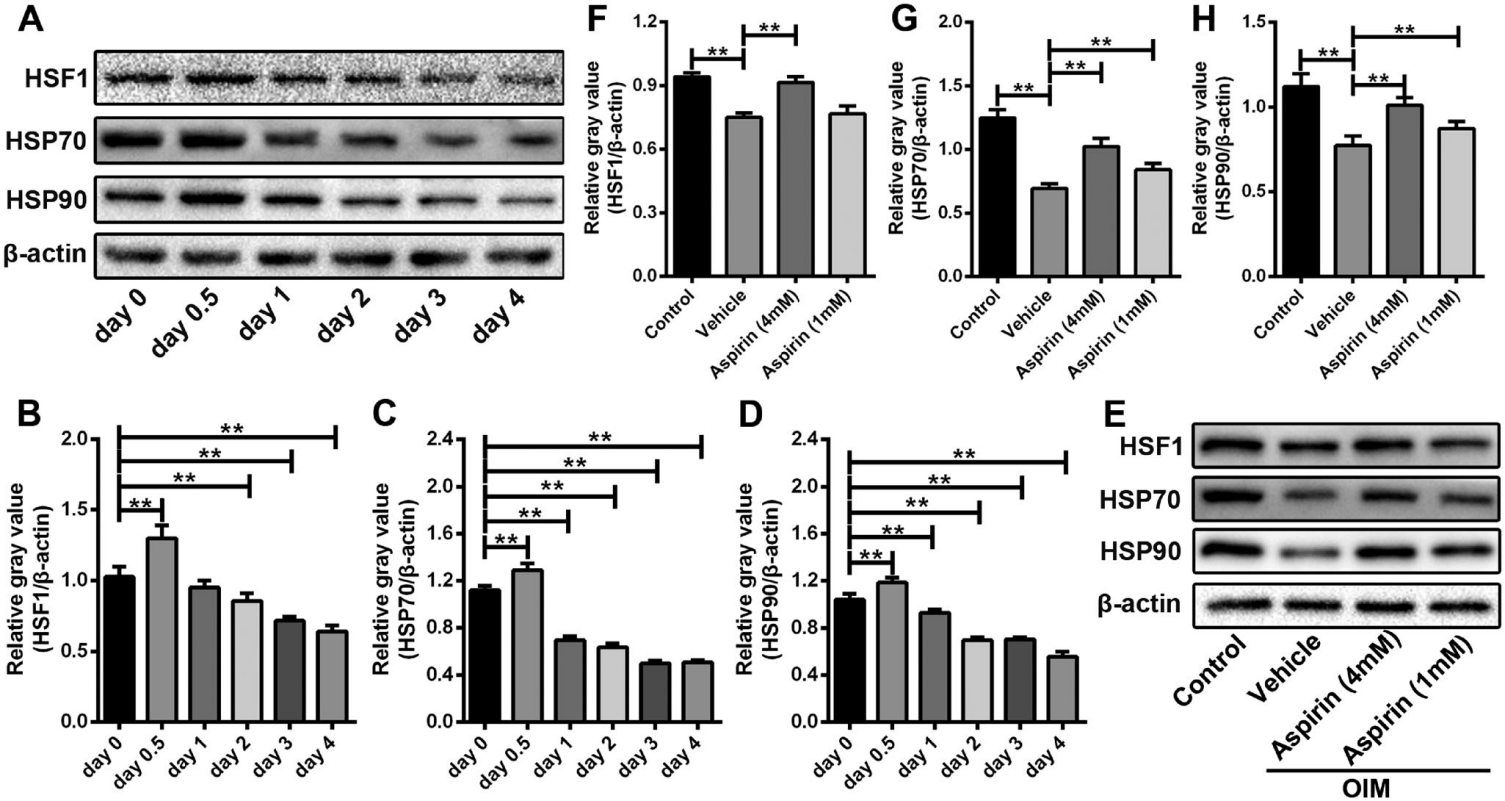
Aspirin enhanced the heat shock response in the calcification process of VSMCs. (A) The expression of heat shock proteins in the process of VSMC calcification. Western blot assay was used to detected protein expression of HSF1, HSP70 and HSP90 in VSMCs after different-time calcification induction. β-Actin was used as loading control. (B–D) Grey values analysis of immune bands in western blot assay. *n* = 3 in each group. **p < 0.01. (E) The effect of aspirin on expression of heat shock proteins in VSMCs after four-day culture in OIM. Western blot assay was used to detect protein expression of HSF1, HSP70 and HSP90 in VSMCs. β-Actin was used as loading control. (F–H) Grey values analysis of immune bands in western blot assay. *n* = 3 in each group. **p < 0.01.

To investigate the change of heat shock response in the process of VSMC calcification, the expression of HSF1, HSP70 and HSP90 was examined in VSMCs at different calcification induction time. HSF1 was slightly upregulated in the first 12 h under calcification induction, and then was constantly downregulated in the following days. Similarly, the expression of HSP70 and HSP90 showed the same changes (Figure 3(A–D)). Then, VSMCs were treated with 1 or 4 mmol/L aspirin during the process of calcification induction. On the 4th day, treatment of aspirin restored the protein expression of HSF1, HSP70 and HSP90, especially with the concentration of 4 mmol/L (Figure 3(E–H)). Although siRNA-mediated HSF1 inhibition did not aggravate the process of VSMC calcification, it could obviously offset the anti-calcification effect of aspirin (Figure S1).

### Inhibition of HSP70 counteracted the anti-calcification effect of aspirin on VSMCs

**Figure 4.**
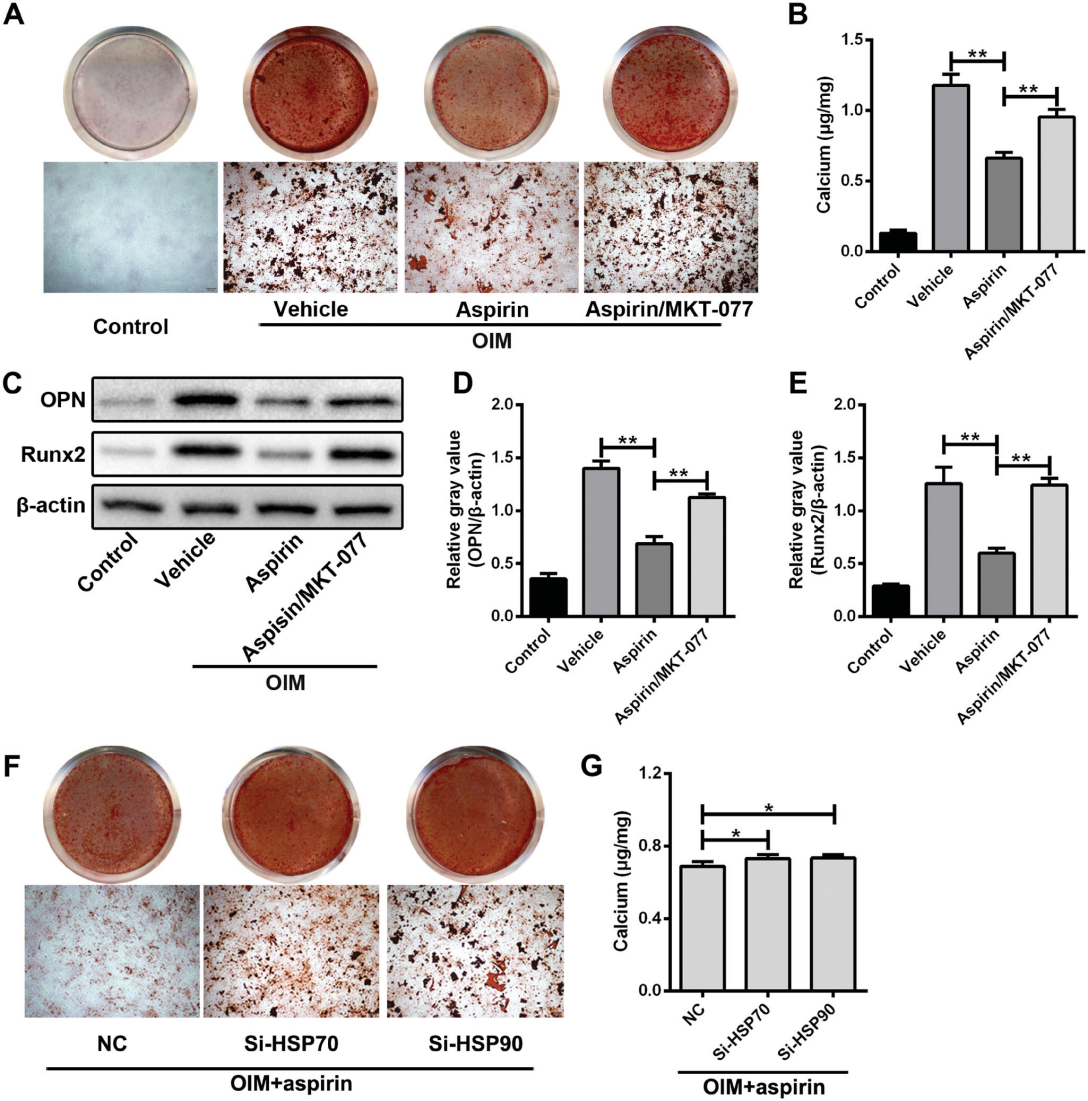
Inhibition of HSP70 counteracted the anti-calcification effect of aspirin on VSMCs. (A) The effect of HSP70 inhibitor MKT-077 on aspirin alleviating VSMC calcification. Alizarin Red S assay was applied to detect calcium nodules in VSMCs after 10-day culture in OIM. (B) The effect of MKT-077 on aspirin reducing intracellular calcium concentration in VSMCs after 10-day culture in OIM. *n* = 5 in each group. **p < 0.01. (C) The effect of MKT-077 on aspirin downregulating osteogenic marker genes expression in VSMCs after four-day culture in OIM. β-Actin was used as loading control. (D, E) Grey values analysis of immune bands in western blot assay. *n* = 3 in each group. **p < 0.01. (F) The effect of HSP70/HSP90 siRNA on aspirin alleviating VSMC calcification. The calcium nodules were detected by Alizarin Red S assay in VSMCs after 10-day culture in OIM. (G) The effect of HSP70/HSP90 siRNA on aspirin reducing intracellular calcium concentration in VSMCs after 10-day culture in OIM. *n* = 5 in each group. *p < 0.05.

To further prove whether aspirin reduced the calcification of VSMCs by enhancing the heat shock response, the small molecule compound (MKT-077) was used to inhibit the activity of HSP70. Results from Alizarin Red S staining and intracellular calcium concentration assays indicated that inhibition of HSP70 by MKT-077 could greatly abolish the anti-calcification effect of aspirin in VSMCs, demonstrated by more calcium nodules and higher calcium concentration in aspirin/MKT-077 group (Figure 4(A,B)). The expression of OPN and Runx2 was also increased in VSMCs from aspirin/MKT-077 group, compared with that from aspirin group (Figure 4(C–E)). Results from Alizarin red S staining and intracellular calcium concentration assays further confirmed that inhibition of HSP70 by siRNA interference could partly abolish the anti-calcification effect of aspirin (Figure 4(F–G)). However, MKT-077 alone treatment had no significant effect on the calcified nodule formation and intracellular calcium accumulation (Figure S2).

### Inhibition of HSP90 reduced the anti-calcification effect of aspirin on VSMCs

**Figure 5.**
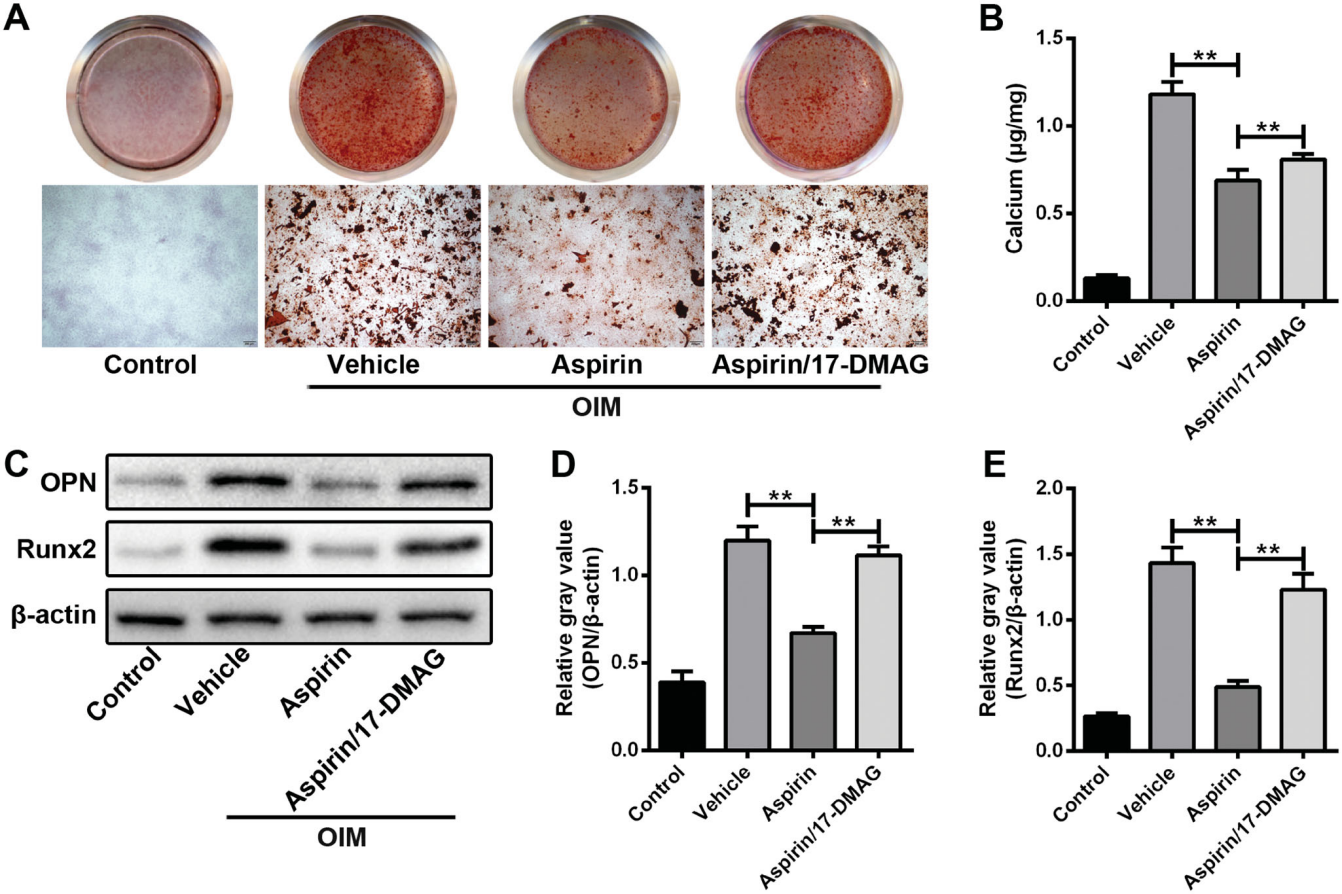
Inhibition of HSP90 reduced the anti-calcification effect of aspirin on VSMCs. (A) The effect of HSP90 inhibitor 17-DMAG on aspirin alleviating VSMC calcification. The calcium nodules were detected by Alizarin Red S assay in VSMCs after 10-day culture in OIM. (B) The effect of 17-DMAG on aspirin reducing intracellular calcium concentration in VSMCs after 10-day culture in OIM. *n* = 5 in each group. **p < 0.01. (C) The effect of 17-DMAG on aspirin downregulating osteogenic marker genes expression in VSMCs after four-day culture in OIM. β-Actin was used as loading control. (D, E) Grey values analysis of immune bands in western blot assay. *n* = 3 in each group. **p < 0.01.

Next, 17-DMAG, an HSP90 inhibitor, was used to explore the role of HSP90 in aspirin alleviation of calcification process in VSMCs. The anti-calcification effect of aspirin in VSMCs was significantly attenuated by 17-DMAG treatment, manifested as more calcium nodules (Figure 5(A)), higher calcium concentration (Figure 5(B)) and higher expression of OPN and Runx2 (Figure 5(C–E)). Results from Alizarin red S staining and intracellular calcium concentration assays confirmed that siRNA-mediated HSP90 inhibition could partly abolish the anti-calcification effect of aspirin (Figure 4(F–G)). However, 17-DMAG alone treatment had no significant effect on the calcified nodule formation and intracellular calcium accumulation (Figure S2). These results further revealed that aspirin relieved the calcification of VSMCs partially through heat shock response.

## Discussion

In the present study, we found that aspirin could alleviate the progress of VSMC calcification by enhancing the heat shock response. Inhibition of HSP70 or HSP90 could partially abolish the anti-calcification effect of aspirin. Therefore, we inferred that aspirin could be applied as a potential drug alleviating vascular calcification.

The dosages of aspirin used to stimulate VSMCs ranged from 10 μmol/L to 10 mmol/L in recent reports (Loppnow et al. 2011; Sung and Choi 2011; Sanada et al. 2016). We found that 1 mmol/L aspirin had a weaker efficacy on alleviating calcification of VSMCs, which may be related to lower concentration of drug. To explore the effect of aspirin on VSMC calcification, we also set a group of VSMCs treated with 10 mmol/L aspirin. However, treatment of aspirin in this concentration caused a large number of deaths of VSMCs, which might be possibly attributed to relatively low pH value affecting cell viability. Although aspirin of 4 mmol/L could produce a significant anti-calcification effect at the cellular level, the drug concentration in medium might be exceeded the maximum blood concentration that human body can withstand. Furthermore, haemorrhage is one of dangerous side effects of aspirin treatment, even in small dosages. Therefore, it is potential to investigate the concrete mechanism of aspirin on alleviating calcification and downstream targets may effectively avoid the side effects of high-concentration aspirin in the clinical practice.

HSPs are usually considered as protective mediators for vascular wall in physiological conditions, which function by regulating misfolded proteins and anti-inflammatory effects (Kilic and Mandal 2012). Studies have shown a significant effect of HSP70 on preventing and inhibiting VSMC calcification. Furthermore, the low expression of HSP70 in the arterial wall is associated with an increased risk of vascular calcification (Lu et al. 2012). However, it was also reported that extracellular HSP70 could promote BMP2/4-induced cell condensation and osteogenic differentiation in calcifying vascular cells by binding with matrix GLA protein (Yao et al. 2009). Application of different methods on inducing cell calcification might be the main reason for this controversial result. Second, the roles and mechanisms of extracellular and endogenous HSP70 might be also different. Administration of extracellular HSP70 might affect multiple signalling pathways in VSMC by activating certain receptors on the plasma membrane. Meanwhile, the dosages of extracellular HSP70 might be related to its effect on VSMC calcification.

Heat shock is the universal response of eukaryotic cells to environmental stimuli. Under normal conditions, HSF1 is maintained in an inactive state, and HSPs are expressed at low levels. The efficient synthesis of HSPs would be rapidly activated upon a nonlethal stimulus, but would be declined under continuing increase of stress exposure which in turn activates apoptosis mechanisms (Lanneau et al. 2008). The present study also found that the expression of HSP70 and HSP90 was upregulated in the first 12 hours of calcification induction, but maintained in low levels in the following days, which suggested heat shock response in VSMCs was not continuously activated during the induction of calcification. It might be the main reason why MKT-077 alone or 17-DMAG alone treatment had no significant effect on VSMC calcification.

Aspirin was first used as one of several conventional therapies for anti-inflammation, as the mechanism of aspirin mainly mediated by acetylating COX enzymes, thereby inhibiting the production of prostaglandin, prostacyclin and thromboxane (Patrono and Rocca 2012). Its effects on a variety of diseases such as atherothrombosis, cancer, pre-eclampsia and mental disease were found subsequently (Hybiak et al. 2020), and in this process its multiple downstream channels had been further explored, not just limited to Cox system. High dosages of aspirin can activate monophosphate-activated protein kinase (AMPK) pathway in cells, thereby reducing plasma lipid and increasing utilization of fat, offering great potential in nutritional and metabolic disorders (Hawley et al. 2012; Guigas and Viollet 2016). In addition, aspirin can bind with PPARα to induce the expression of transcription factor EB and increase intracellular lysosome biogenesis and autophagy, which was helpful for plaque clearance (Chandra et al. 2019).

## Conclusions

In this study, aspirin relieved the calcification of VSMCs partially through HSP70- or HSP90-mediated heat shock response. These findings expanded the understanding of aspirin pharmacology, and suggested local induction expression of HSPs as a potential therapeutic strategy for the prevention and reversal of vascular calcification.

## Supplementary Material

Supplemental Material
