# Supplementary material for: Aspirin relieves the calcification of aortic smooth muscle cells by enhancing the heat shock response
Source: Pharm Biol. 2021 Nov 30;60(1):17–24. doi: 10.1080/13880209.2021.2007268 (PMC8635617; doi:10.1080/13880209.2021.2007268)
Supplement: Supplemental Material [file IPHB_A_2007268_SM6544.docx]

**Supplemental data**

**Figure S1**


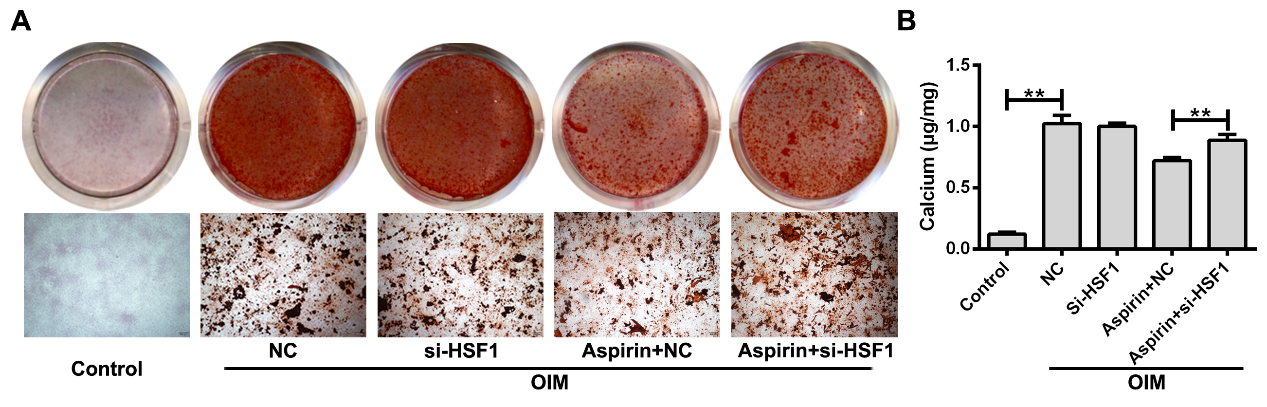


Figure S1 Inhibition of HSF1 by siRNA reduced the anti-calcification effect of aspirin on VSMCs. A, The effect of HSF1 siRNA on aspirin alleviating VSMC calcification. The calcium nodules were detected by Alizarin Red S assay in VSMCs after 10-day culture in OIM. B, The effect of HSF1 siRNA on aspirin reducing intracellular calcium concentration in VSMCs after 10-day culture in OIM. n=5 in each group. **P<0.01.

**Figure S2**


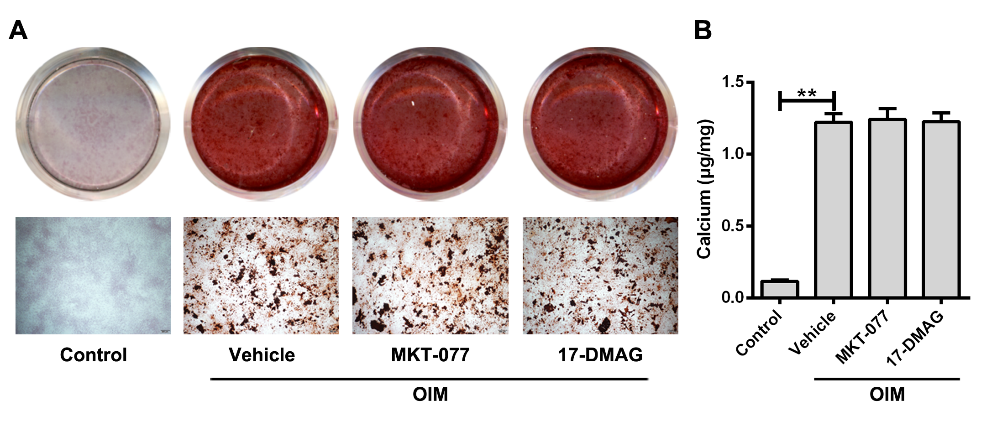


Figure S2 MKT-077 alone and 17-DMAG alone treatment had no significant effect on VSMC calcification. A, The effect of MKT-077 or 17-DMAG on VSMC calcification. The calcium nodules were detected by Alizarin Red S assay in VSMCs after 10-day culture in OIM. B, The effect of MKT-077 or 17-DMAG on intracellular calcium concentration in VSMCs after 10-day culture in OIM. n=5 in each group. **P<0.01.
